# Potential Protective Factors for Allergic Rhinitis Patients Infected with COVID-19

**DOI:** 10.3390/cimb46070395

**Published:** 2024-06-28

**Authors:** Jiaoyue Dong, Dingyuan Su, Binbin Zhao, Jiayang Han, Mengjie Tu, Kaifeng Zhang, Fengling Wang, Yang An

**Affiliations:** 1 Department of Biochemistry and Molecular Biology, School of Basic Medical Sciences, Henan University, Kaifeng 475004, China; 2 Henan Provincial Engineering Center for Tumor Molecular Medicine, Kaifeng Key Laboratory of Cell Signal Transduction, Henan University, Kaifeng 475004, China

**Keywords:** COVID-19, allergic rhinitis, ACE2 receptor

## Abstract

At the beginning of the 2019 coronavirus disease (COVID-19) pandemic, airway allergic diseases such as asthma and allergic rhinitis (AR) were considered as risk factors for COVID-19, as they would aggravate symptoms. With further research, more and more literature has shown that airway allergic disease may not be a high-risk factor, but may be a protective factor for COVID-19 infection, which is closely related to its low-level expression of the ACE2 receptor and the complex cytokines network as underlying molecular regulatory mechanisms. In addition, steroid hormones and age factors could not be ignored. In this review, we have summarized some current evidence on the relationship between COVID-19 and allergic rhinitis to highlight the underlying mechanisms of COVID-19 infection and provide novel insights for its prevention and treatment. The key findings show that allergic rhinitis and its related molecular mechanisms may have a protective effect against COVID-19 infection.

## 1. Introduction

Since the declaration of COVID-19 as a pandemic by the World Health Organization (WHO), the number of confirmed cases worldwide has surpassed 760 million, with over 6.9 million deaths as of 12 July 2023 (https://covid19.who.int./). Initially, asthma and allergic rhinitis were considered as risk factors due to their association with severe COVID-19 [1,2]. However, along with thorough research, more and more evidence demonstrates that COVID-19 has a low incidence among patients with allergic diseases including asthma and allergic rhinitis, leading to discussions on the potential protective effect on severe COVID-19 provided by these allergic diseases [3,4].

Allergic rhinitis (AR) is a prevalent nasal disease, affecting a substantial proportion of the global population (10% to 40%) [5]. Several studies have suggested that individuals with AR may exhibit lower susceptibility to COVID-19, milder early symptoms, and less severe lung disease. Additionally, these patients may experience faster negative conversion of viral nucleic acid [6,7,8]. The purpose of this study is to explore the possible protective factors of AR patients upon COVID-19 infection. In view of the seriousness of the COVID-19 epidemic and the challenge to global public health, we pay attention to the special group of AR patients, because they may have different reactions to the virus due to the specific state of the immune system. Through a review and retrospective of previously reported literature, this study tries to reveal the mechanism behind these protective factors and hopes to provide a new perspective and strategy for the prevention and treatment of COVID-19.

Although lots of studies focus on COVID-19 patients in general, there are relatively few studies concerned about AR patients. By focusing on AR patients, this study reveals the potential protection mechanism of this special group in virus infection. We not only pay attention to the pathophysiological process of COVID-19, but also explore the protective factors that may reduce the risk of infection or the severity of the disease in AR patients, and provide new ideas for clinical prevention and treatment. This study comprehensively analyzes the research data in different fields to understand the infection mechanism of COVID-19 in AR patients. By revealing the possible protective factors and mechanism of AR patients, this study is expected to provide scientific basis for developing new treatment methods and drugs for COVID-19, so as to prevent and control the spread of the epidemic more effectively.

Based on PubMed, this review searched the previously reported studies related to allergic rhinitis since the epidemic of COVID-19, with the keywords ‘COVID-19 and allergic rhinitis’ and ‘SARS-CoV-2 and allergic rhinitis’.

## 2. Allergic Rhinitis (AR)

As a serious global health issue, allergic rhinitis is increasingly prevalent and imposes a considerable medical and socioeconomic burden. It is an allergic disease with multi-molecular regulation and cell involvement. In allergic rhinitis, nasal mucosal dendritic cells (MDCs) are capable of capturing allergens and presenting them to CD4+ T cells, thereby activating ILC2s through the IL-33/ST2 pathway to generate type 2 cytokines [9]. In contrast to healthy individuals, patients with allergic rhinitis exhibit a distinct subset of memory T cells related to allergic respiratory disease, known as pathogenic Th2 (Th2A) cells [10,11]. The elevated expression of epithelial cell-derived cytokine receptors leads to the increased production of IL-5 and IL-9 secreted by Th2A cells [12]. Unlike Th2 and Th2A cells, TFH cells secrete IL-4 and IL-13 and have an ability to prime B cells for allergen-specific IgE production [13,14]. Regulatory B cells, a subtype of suppressive B cell, have been demonstrated to secrete IL-10 in the regulation of allergic respiratory inflammation [15].

## 3. Effects of COVID-19 Infection on AR Patients

### 3.1. ACE2 and TMPRSS2

Angiotensin-converting enzyme 2 (ACE-2) is mainly expressed in type II lung cells and ciliated cells [16]. It is the main receptor for COVID-19 to enter the host cell through its structural spike glycoprotein. The transmembrane protease serines (TMPRSS2), expressed in human respiratory epithelial cells, can activate the S protein to enhance the entry of viruses into the primary target cells and their spread in the infected host [17,18,19]. COVID-19 enters the host cell through the binding of its spike protein to ACE2, and this process is facilitated by TMPRSS2 of the host, which cleaves the spike protein into S1 and S2 fragments, thereby enabling cell membrane fusion [20,21]. The expression of quantitative trait loci (EQTL) for ACE2 and TMPRSS2 varies in populations around the world [22], which may be due to population differences in COVID-19 infection. Other molecules, including CD147 and CD26, may also act as COVID-19 receptors to help it enter into human cells [23,24]. Additionally, the expression regulation of ACE2 and TMPRSS2 is mainly determined by T2 inflammation and virus-induced interferon inflammation [22].

It has been evidenced that the expression of ACE2 in nasal and bronchial epithelial cells of patients afflicted with AR or allergic asthma is significantly decreased, which is possibly related to allergen exposure, allergen sensitization, and a high IgE level [25,26,27]. Besides, compared with healthy individuals, AR patients have a higher level of TMPRSS2 in their nasal cavity [28]. Therefore, as the portal of virus invasion, the low expression of ACE2 in respiratory epithelial cells of AR patients can reduce the possibility of infection and deterioration of COVID-19 in AR patients, thus counteracting the potential adverse consequences caused by the increase in TMPRSS2 [19,27,29,30].

### 3.2. Th2 Cytokine

**Figure 1:**
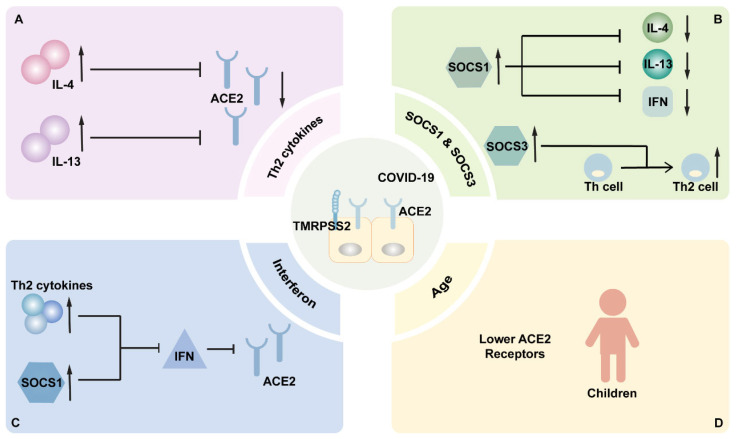
Major cytokines and factors exert influences on antivirus defense by reducing ACE2 expression in AR patients. (**A**) Th2 cytokines, including IL-4 and IL-13, can reduce ACE2 expression. (**B**) The high expression of SOCS1 or SOCS3 in AR patients can reduce the interferons level and promote Th2 cell differentiation, ultimately reducing the ACE2 level in the nasal epithelium. (**C**) Th2 cytokines and SOCS1 can negatively control the production of interferons, which may reduce ACE2 expression in AR patients. (**D**) The expression of ACE2 can change with age, and children have relatively lower ACE2 receptors.

Cytokines play a significant role in AR, forming a cytokine network to coordinate specific process of AR. AR is generally caused by an overreaction of T-helper 2 lymphocytes (Th2), which are considered to be an important source of Th2 cytokines [31,32]. Type 2 cytokines mainly include IL-4, IL-5, IL-9, and IL-13 (Table 1). These cytokines, produced by eosinophils, mast cells, basophils, and Th2, play a major role in regulating antibody transformation, humoral immunity, and tissue damage repair [33]. Th2 cytokines are defined to allow bacterial invasion as they can inhibit epithelial barrier proteins and antimicrobial proteins [34,35]. Meanwhile, in AR patients infected with COVID-19, Th2 cytokines have been speculated to play a protective role [4,22], as they are able to decrease ACE2 expression, allowing individuals to experience a better outcome after COVID-19 infection [22,36] (Figure 1). Additionally, IL-4 and IL-13 were found to reduce ACE2 expression in Vero E6 cells, a primate renal epithelial cell line [37] (Table 1). IL-13 is proved to significantly down-regulate the expression of ACE2 in secretory cells (Figure 1). Meanwhile, IL-13 is able to induce the up-regulation of TMPRSS2 in mucus secreting cells (Table 1), stimulating mucification and inflammatory epithelial cell hyperplasia, whereas the effect of up-regulated TMPRSS2 would be offset by the decreased ACE2 expression [22].

### 3.3. SOCS1 and SOCS3

In general, cytokines are negatively regulated by members of the suppressor of cytokine signaling (SOCS) family through multiple mechanisms [38]. Most studies mainly focus on SOCS1 and SOCS3, which are highly expressed in patients afflicted with asthma, allergic rhinitis, psoriasis, and allergic dermatitis [39,40]. In particular, they negatively regulate cytokine signaling through different mechanisms and may become new targets for therapeutic drug development. SOCS1 is a negative regulator of Th2 cytokines and overexpressed in AR patients. In bronchial epithelial cells after viral infection, SOCS1 expression can be induced by Th2 cytokines and proinflammatory cytokines [41,42]. SOCS1 suppresses IL-4-STAT6-mediated gene expression in macrophages, thereby inhibiting IL-4 signal transduction, which is important for regulating macrophage activation (Figure 1). Given that the deficiency of STAT6 regulation is related to the pathogenesis of certain diseases, controlling STAT6 activity appears to be meaningful in preventing some chronic inflammatory reactions [22,43]. SOCS1 could also inhibit IL-13 signaling by inhibiting STAT6 phosphorylation in bronchial epithelial cells, supporting its crucial role in regulating Th2 cytokines involved in COVID-19 infection [25,41] (Figure 1). Furthermore, SOCS1 can impair the level of interferon (IFN) expression to some degree [44] (Figure 1). SOCS3 plays a regulatory role in a variety of allergic diseases, and the severity of allergic diseases is closely related to its selective expression in Th2 cells: a high expression of SOCS3 drives Th cells to differentiate into Th2 cells [43] (Figure 1). During AR development, SOCS3 has been implicated in mucus secretion and has the potential to be a major cytokine regulator [45]. However, some experiments have shown that SOCS3 is the main cytokine regulatory factor in the pathogenesis of AR, while SOCS1 may not play an important role in this process [46]. Therefore, the high expression of SOCS1 and SOCS3 in AR patients is able to reduce viral invasion by reducing the interferon level, facilitating Th2 cell differentiation, and ultimately reducing the ACE2 level in the nasal epithelium [22,44]. In addition, some studies suggest that SOCS-3 and its ligases may become new targets for the development of allergy-related therapeutic drugs [38,43].

### 3.4. IFN

Type I interferon (IFN-α/IFN-β) is an antiviral cytokine produced by a variety of cells in response to viral infection and the binding of various pattern recognition receptors (PRRs), constituting the first line of defense against viruses [47,48]. Type I IFN-α and IFN-β signal transmits through forming a complex of IFN-α receptor IFNAR1 and IFNAR2, while type III IFN-γ acts through the interaction of the IL-10 receptor β chain and IFN-γ receptor α chain [49]. Type I and type III IFNs are induced during respiratory viral infections. There is increasing evidence that suggests that type III IFNs are mainly responsible for protecting the respiratory tract from viral invasion and play a crucial role in local antiviral innate immunity [50,51,52]. Accumulating evidence has proved that severe infection and the persistence of Th2 inflammation are caused by the lack of regulation of type I interferon [53,54,55].

**Figure 2:**
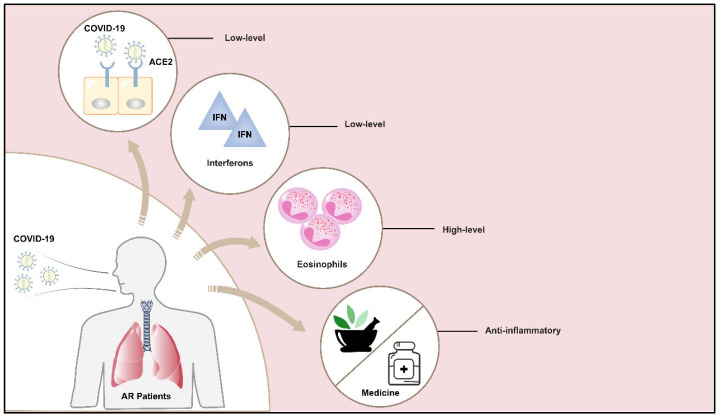
AR patients have lower levels of ACE2 receptors or interferons, but higher levels of eosinophils, compared with healthy people. In addition, some AR patients also receive inhaled corticosteroids (ICSs) and traditional Chinese medicine treatment, which may reduce the possibility of virus invasion and improve prognosis for AR patients.

Viral infection destroys the epithelial barrier and leads to the production of the pro-Th2 cytokines IL-33 and IL-25. These cytokines act on type 2 innate lymphoid cells (ILC2s), dendritic cells (DCs), and Th2 cells to produce the Th2 cytokines IL-13, IL-4, and IL-5 [56,57,58]. IL-4 and IL-13 can stimulate B cells to produce more IgE antibodies, and IgE will then cross-link with FcεRI, leading to a decrease in TLR3 expression. This reduction will eventually decrease the secretion of type I interferon used for virus defense, leading to the increase in rhinovirus replication [47,56,57]. Therefore, the level of interferon in the nasal epithelial cells of AR patients is lower than that of normal nasal epithelial cells [58]. Intriguingly, with further research, it was found that high levels of interferon are more likely to produce virus carriage [59]. This may also be another factor that makes AR patients less susceptible to COVID-19 infection. In addition to Th2 cytokines, levels of interferon also have a strong effect on ACE2 expression (Figure 1). In detail, ACE2 levels were 1.7-times higher in the high interferon group than that in the low interferon group, and IFN-α, IFN-γ, and TNF-α also up-regulate the expression of ACE2 mRNA, which makes viral invasion more easy [22,25]. Therefore, lower interferon levels in AR patients may be more beneficial in reducing viral invasion (Figure 2).

Another interesting finding comes from studies analyzing the expression of SOCS1, a regulator that negatively controls the production of type I and type II interferons, which is induced by Th2 cytokines and specific respiratory viruses [44,60]. In murine models, it has been shown that SOCS1 plays a direct role in suppressing interferon production following RV infection. Increased expression of SOCS1 has been found in cases of interferon deficiency. This finding may support the idea that SOCS1 is an important inhibitor of interferon signaling in vivo [40,44,61] (Figure 1).

### 3.5. Eosinophils

Blood eosinophil level is a powerful index for predicting airway T2 inflammation [61]. Th2 cytokines in AR patients not only participate in IL-4-induced IgE conversion and IL-5-induced eosinophil induction and activation, but also in IL-13-induced eosinophilic airway inflammation. Additionally, they are involved in the periairway fibrosis induced by double regulatory proteins and aseptic inflammation induced by Charcot–Leyden crystals [62,63]. In particular, IL-5 is the main stimulating factor for the differentiation, recruitment, activation, and survival of eosinophils [64]. Therefore, the higher eosinophil level in AR patients may be associated with Th2 cytokines.

In a recent study of 85 fatal COVID-19 subjects, it was found that 81.2% of them had significantly low levels of eosinophil in their blood [65,66]. The low level of eosinophils is a prognostic indicator for a more severe COVID-19 disease [67]. Eosinophils play an important role in accelerating virus clearance and antiviral host defense [68]. The presence of eosinophilia in asthma patients also has an obvious protective effect [30]. Therefore, the ability of high-level eosinophils to resist virus infection in AR patients may explain the low prevalence of COVID-19 in AR patients (Figure 2).

### 3.6. Age

The expression of ACE2, a receptor of COVID-19, can change with age [69] (Figure 1). As age increases, the content of the ACE2 protein in olfactory epithelial cells also increases along with the expression of two host receptors of COVID-19. This may explain why older humans are more likely to be infected with COVID-19 [70]. Thus, the susceptibility and severity of COVID-19 increase with age [71]. Additionally, AR patients are often younger than those with other complications. This age difference once again suggests that there may be a relationship between the level of ACE2 receptors and susceptibility to COVID-19 infection in AR patients.

## 4. Effects of Drug Therapy on AR Patients

### 4.1. ICSs Produce Protective Effects on Allergic Patients by Reducing the Expression of ACE2 and TMPRSS2

Due to their powerful anti-inflammatory effects, corticosteroids play a crucial role in the treatment of respiratory allergic diseases, including AR, which can alleviate the related symptoms of AR [72,73]. The commonly used drugs are inhaled corticosteroids (ICSs) and intranasal corticosteroids (INCSs). Several published studies have shown that it is beneficial for AR patients infected with COVID-19 to continue inhaling the recommended dose of corticosteroids [74,75], indicating that ICSs have a protective effect on AR patients afflicted with COVID-19. Corticosteroids not only have a strong anti-inflammatory effect, but also have an antiviral effect, which is mainly reflected in inhibiting virus invasion, replication, and transcription. ICSs have been proved to reduce the expression of ACE2 and TMPRSS2 in the sputum in a dose-dependent manner within a certain range. However, triamcinolone acetonide (TA) does not have the same effect [69]. Subsequently, some studies found that ICSs may lead to the down-regulation of ACE2 in the lungs by mediating the inhibition of type I interferon [76], which is of great significance for reducing the susceptibility of patients to COVID-19 and alleviating clinical symptoms. Additionally, it has been proven that the ICS cyclosonide can specifically inhibit RNA replication and transcription of the coronavirus in some experiments and has concentration-dependence and low cytotoxicity [77]. Importantly, some studies have demonstrated that inhaling cyclosonide has fewer clinical adverse reactions and is relatively safe [78,79].

### 4.2. Traditional Chinese Medicine Can Inhibit Interleukin-6 to Exert Antiallergic and Antiviral Treatment

Currently, a study has proved that a Chinese herbal medicine formula based on *Houttuynia cordata* Thunb. can alleviate the symptoms of AR and also exhibit antiviral properties. As a result, some AR patients who use this kind of Chinese herbal medicine are more likely to reduce their susceptibility to COVID-19 [80].

Studies have shown that the increase in cytokines in the sinus mucosa of AR patients is associated with an increase in NF-κB expression, and the NF-κB pathway triggers the amplification of IL-6 [81]. Intriguingly, IL-6 acts through a NF-κB positive feedback loop mediated by the NF-κB pathway and plays an important role in amplifying the inflammatory response in a mechanism study of COVID-19-induced cytokine storm [82]. Therefore, it may be possible to treat inflammation induced by COVID-19 and alleviate symptoms in AR patients by specifically inhibiting IL-6.

A traditional Chinese herbal formula (ZYF) based on *Scutellaria baicalensis* and *Houttuynia cordata* has been shown to have the dual effects of being antiviral and alleviating allergic symptoms in AR patients (Table 2). *Houttuynia cordata* has been shown to inhibit FcεRI-mediated signaling pathways in mast cells, such as NF-κB activation, which helps repair damage to the nasal mucosa. Baicalin can significantly inhibit IL-6 secretion from murine macrophages and then exert anti-inflammatory effects (Table 2). These two drugs are the main constituents of ZYF [80].

Additionally, the treatment of human recombinant soluble ACE2 (hrsACE2) significantly inhibits the growth of Vero cells infected with COVID-19 and the infection of COVID-19 in human blood vessels and kidney organs [83]. Camostat, a serine protease inhibitor, prevents COVID-19 from entering human primary lung cells [29] (Table 2). Similarly, nafamostat inhibits the membrane fusion of the S protein during COVID-19 infection [84] (Table 2). Azelastine is thought to interfere with the interaction of the spike glycoprotein and ACE2 by fixing the receptor in a closed form, and the application of azelastine nasal spray can accelerate the clearance of the virus [85,86] (Table 2). Allergic Rhinitis Nose Drops (ARNDs) can not only inhibit pseudovirus infection and destroy the combination of ACE2 and the spike protein (Delta), but also reduce the inflammatory reaction after infection, which may lead to better prognosis and lower risk of pulmonary fibrosis [87] (Table 2). A recent study has found that sublingual immunotherapy (SLIT) is not only the most effective method for treating AR but also has a certain protective effect on COVID-19 infection [88].

**Table 2:** Antiviral mechanisms of drugs on COVID-19 infection.

Treatment	Type of Drug	Antiviral Mode	References
Ciclesonide	inhaled corticosteroid	suppresses COVID-19 replication and	[77]
		reduces the expression of ACE2 and TMPRSS2	
Camostat	serine protease inhibitor	blocks TMPRSS2 activity	[29]
Nafamostat	serine protease inhibitor	inhibits the S protein membrane fusion during COVID-19 infection	[84]
Azelastine	antihistamine	accelerates the clearance of virus	[85]
		blocks spike protein/ACE2 interaction	[86]
HrsACE2	human recombinant soluble ACE2	binds ACE2 receptor	[83]
ARND	Chinese medicine nasal spray	blocks spike protein/ACE2 interaction and down-regulates the expression of proinfammatory cytokines	[87]
ZYF	Chinese medicine	repairs pathological changes in nasal mucosa, decreases the activation of IL-6, and blocks spike protein/ACE2 interaction	[80]

### 4.3. Patients with Allergic Rhinitis May Experience a Stronger Protective Effect after Vaccination

Intriguingly, the protective humoral immunity of AR patients to the COVID-19 vaccine is enhanced, which is related to the increase in circulating Tfh2 cells and protective antibodies after vaccination. The serological reaction of AR patients is stronger than that of healthy controls, showing a higher level of neutralizing antibodies [89], which are more conducive to blocking viral infections in the human body. The latest research indicates that receiving at least two doses of the COVID-19 vaccine has a protective effect on subsequent allergic diseases such as AR and reduces the risk of post COVID-19 allergic diseases. Therefore, vaccination is advocated as one of the strategies to prevent patients from post COVID-19 diseases [90].

## 5. Discussion

The immune response mechanism of patients with AR mainly involves humoral, cellular, and mucosal immunity. These immune mechanisms play important roles in the pathogenesis of AR and may affect the resistance of AR patients to COVID-19.

Humoral immunity: the immune system of AR patients will produce specific antibodies against allergens after contact with allergens. These antibodies circulate in body fluids and may combine with allergens to form immune complexes. These immune complexes can activate the complement system and release inflammatory mediators, leading to inflammation and the swelling of the nasal mucosa [91]. However, this humoral immune response may also affect the resistance of patients to COVID-19. On the one hand, the immune system of AR patients has been trained to some extent when dealing with allergens, and may be more sensitive and active, so as to better identify and deal with the COVID-19 virus [89]; on the other hand, the formation of the immune complex may interfere with the combination of COVID-19 virus and host cells, reducing the risk of virus infection [92].

Cellular immunity: cellular immunity of AR patients plays an important role in allergic reaction. Immune cells such as eosinophils and mast cells play a key role in the occurrence and development of allergic reactions. These cells can release inflammatory mediators, such as histamines and leukotrienes, causing inflammation and the swelling of the nasal mucosa [93]. Additionally, cellular immunity also plays an important role in COVID-19 infection. In particular, the cellular immunity of AR patients may be more active, which is helpful to remove the infected virus [94]. However, excessive cellular immune response may also lead to tissue damage and an increased inflammatory response, thus affecting the severity of COVID-19 [95].

Mucosal immunity: the nasal mucosal immune system of AR patients will also undergo a series of changes when dealing with allergens. These changes may include the destruction of nasal mucosa epithelial cells, the release of inflammatory mediators, and the infiltration of immune cells, which may lead to the activation of the immune response of the nasal mucosa and affect the resistance of patients to COVID-19. On the one hand, the activated mucosal immune system may better identify and remove the infected virus [96]; on the other hand, excessive mucosal immune response may also lead to increased nasal mucosal inflammation and affect respiratory function [97]. Previous studies mainly focused on one aspect of the immune response mechanism of AR patients, such as humoral immunity or cellular immunity, but this study adopted a more comprehensive perspective, taking humoral immunity, cellular immunity, and mucosal immunity into consideration, and providing a more comprehensive framework for understanding the immune response of AR patients. Most previous studies have not linked the immune response of AR patients with COVID-19 infection. Under this background, this study conducted an in-depth exploration and analyzed how the immune response mechanism of AR patients affected their resistance to COVID-19, which provided new ideas for prevention and treatment. Previous studies focused on the static state of immune response in AR patients, but this study not only analyzed the static state but also tracked the dynamic changes in these immune responses during COVID-19 infection. The study of this dynamic change is helpful to evaluate the resistance of AR patients to COVID-19 more accurately, and it provides a more accurate basis for the formulation of treatment strategies. Through an in-depth understanding of the immune response mechanism of AR patients, this study provides a scientific basis for optimizing the COVID-19 vaccination strategy for AR patients and formulating personalized treatment plans, which is expected to reduce the risk of COVID-19 infection in AR patients or reduce the severity of the disease.

## 6. Perspective

In general, studies on the relationship between AR and COVID-19 still have controversies and uncertainties, and this may be due to the complexity of these diseases and the diversity of research methods. Future research will further explore the immune mechanisms of AR patients and how these mechanisms affect the resistance of patients to COVID-19. For instance, some studies began to explore the pathophysiological characteristics of COVID-19 in patients with allergic diseases. A recent study focused on the radiopathological features of patients with COVID-19-related mediastinal emphysema and discussed the possible role of Sonic hedgehog and Wnt5a pathways in it [98]. In particular, these pathways may be involved in the process of lung injury caused by COVID-19, and allergic diseases may reduce the severity of COVID-19 by affecting the expression of these pathways. Additionally, another study used a quantitative computed tomography (CT) lung COVID-19 score combined with laboratory markers to predict the rapid progress of patients with COVID-19 pneumonia and monitor the longitudinal changes [99]. This study provides a new tool for evaluating the progress of COVID-19 patients, helps us to better understand how allergic diseases affect the course of COVID-19, and provides us with new ideas for treatment strategies. Through the genetic study of AR–asthma syndrome, researchers can better understand the genetic basis of this disease and how genetic factors affect the immune response and disease manifestations in patients. This may provide new therapeutic targets and preventive strategies. Future research should also pay attention to the influence of environmental factors on AR and COVID-19. By understanding the types of allergens and irritants in different geographical areas, we can better understand how these factors affect the incidence and severity of AR and COVID-19. This may provide us with new ideas for prevention strategies and help us to formulate more effective public health policies. In a word, researchers will better understand the relationship between them and provide a more scientific basis for clinical treatment and prevention.

## Figures and Tables

**Table 1:** Mechanisms of cytokines in AR and COVID-19 infection.

Cytokines	Mechanisms
IL-4	down-regulates ACE2 expression to inhibit COVID-19 replication at transcription level
IL-5	induces eosinophil induction and activation to increase eosinophil numbers
IL-6	mediates a positive feedback loop for NF-κB signaling amplifying IL-6, and induces a cytokine storm
IL-13	down-regulates the expression of ACE2 and induces the up-regulation of TMPRSS2
IL-25	augments type-2 cytokine production
IL-33	acts as a pro-Th2 cytokine and induces Th2 cytokines (IL-13, IL-4, and IL-5)
